# Air Pollution, Foreign Direct Investment, and Mental Health: Evidence From China

**DOI:** 10.3389/fpubh.2022.858672

**Published:** 2022-05-20

**Authors:** Wei Jiang, Yunfei Chen

**Affiliations:** ^1^School of Economics, Qingdao University, Qingdao, China; ^2^School of Economics, Shanghai University, Shanghai, China

**Keywords:** mental health, FDI, air pollution, threshold, CHARLS

## Abstract

Recently, there has been interest in the relationship between mental health and air pollution; however, the results are inconsistent and the contribution of foreign direct investment (FDI) has received little attention. This article studies the effects of air pollution on mental health and the moderating role of FDI based on the China Health and Retirement Longitudinal Study (CHARLS) data in 2015 and 2018 applying the fixed effects panel regression approach and the threshold model. The results show that mental health is adversely affected by air pollution, especially PM_2.5_, PM_10_, sulfur dioxide (SO_2_), carbon monoxide (CO), and nitrogen dioxide (NO_2_). Second, FDI has an alleviating influence on the negative relationship. Third, the effects of air pollution and FDI are heterogeneous based on regional characteristics, including location, medical resource and investment in science and technology, and individual characteristics covering education level, age, income, and physical health. Finally, the threshold effects show that FDI has a moderating effect when it is >1,745.59 million renminbi (RMB). There are only 11.19% of cities exceeding the threshold value in China. When the value of air quality index (AQI) exceeds 92.79, air pollution is more harmful to mental health. Government should actively introduce high-quality FDI at the effective level and control air pollution to improve mental health.

## Introduction

Mental health, as an important public health challenge, has received increased attention, especially during coronavirus disease 2019 (COVID-19) outbreak. Mental health refers not only to cognitive ability and the absence of mental diseases, but also to a good mental state (1). Air pollution is concerned as one of leading ten risk factors for human (2), mainly including particulate matter (PM_2.5_ and PM_10_), carbon monoxide (CO), nitrogen dioxide (NO_2_), sulfur dioxide (SO_2_), and ozone (O_3_). Air pollution can increase the mortality, subjective illness, and medical and psychological problems (3–6). There were 6.67 million deaths worldwide due to air pollution according to the Global Burden of Disease Report 2019. Numerous studies have examined the impacts of air pollution on physical health (7). In recent years, there has been a surge of interest in the effects of air pollution on mental health (8, 9). The incidences of mental illness will increase 6.67%, if the concentration of PM_2.5_ increases a one SD (10).

China, the focus of this study, is one of countries with serious air pollution in the world. A large number of air pollutants are brought by rapid economic growth. In China, 135 cities have exceeded environmental air quality standard, accounting for 40.1% of the total 337 cities according to 2020 China Ecological and Environmental Quality Bulletin. The World Health Statistics 2021 reports that air pollution has caused 112.7 deaths per 100,000 population in China in 2016. The elderly and children, who are more vulnerable to air pollution (11, 12), have attracted considerable scholarly attention (13). Therefore, we study the effects of air pollution on mental health, including memory, cognitive ability, and emotions of middle-aged and elderly residents. A better understanding of different influences of air pollution would help to put forward the corresponding countermeasures and reduce adverse effects on health. The results also have implications for other developing countries.

Although previous studies have shown the relationship between mental health and air pollution, there are some inconsistent findings (14). The answers to the above questions and the moderating role of foreign direct investment (FDI) need to be further studied and judged in the light of China's current situation. In addition, it is challenging to accurately identifying the relationship between mental health and air pollution because of endogenous problems. The first is reverse causality. Mental illness may lower labor supply (15) and make workers less productive (16), which, in turn, also affects air pollution caused by economic activities. The second is measurement error of air pollution, which will bias the estimated results (17). We instrument air pollution using the ventilation coefficient to avoid the endogenous biases (18).

In addition, there has been little discussion about the moderating effect of FDI on this nexus. China has witnessed a rapidly growing economy since the reform and opening-up. A large number of FDI play an important role during the period. China has become the largest country in foreign capital inflows owing to a series of preferential policies. Global FDI plunged by 42% to $859 billion in 2020, while FDI in China bucked the decreasing trend and rose to $163 billion. China has ranked as the largest counties of foreign capital inflow, surpassing the United States for the first time according to the Organization for Economic Cooperation and Development. Economic growth in China owes too much large inflows of FDI and its impacts on environment have gradually become a topic. On one hand, FDI could increase the investment of residents and government in mental health through promoting economic growth and arousing the awareness against pollution. Income level is associated with affordability for health investment, thus influencing mental health. On the other hand, China's government has encouraged more FDI to flow into the high-tech industry in recent years. These FDI supports the “pollution halo” hypothesis, which will also help to alleviate the effect of air pollution. Therefore, we study the influence of FDI to have a better understanding of the influencing mechanism of air pollution on mental health.

This article makes several contributions. First, we use entropy weight method to construct the mental health index, which consists of episodic memory, cognitive ability, and depressive symptoms (Center for Epidemiologic Studies-Depression). According to the definition of the WHO, mental health includes not only the absence of mental diseases, but also a good cognitive ability and welfare state (1). Most studies on mental health refer to only one aspect such as happiness, depression, and life satisfaction (19, 20). Besides mental state, this study also investigates the impact of air pollution on the episodic memory and cognitive ability of residents. Although Shen et al. study the same three indicators as in this article, they used a simple arithmetic average method (7). The entropy method is based on the variation degree to avoid the deviation caused by human factors, which could evaluate mental health more accurately.

Second, prior studies neglected the moderating effect of FDI on the relationship between air pollution and mental health. Whether and how the effects of air pollution on mental health when FDI levels are considered become an empirical question. The role of FDI in the light of China's current situation is uncertain. This article attempts to fill the gaps in the literature.

Third, the effects of air pollution and FDI on mental health can be very diverse in regional and individual characteristics. Moreover, air quality index (AQI), as the measurement indicator of air pollution, is calculated as a composite indicator of *PM*_2.5_, *PM*_10_, *CO*, *NO*_2_, *SO*_2_, and *O*_3_. Different types of air pollution may differ in their characteristics. We study heterogeneity in the effects of air pollution and FDI on mental health not only including the difference of regional and individual characteristics, but also the different types of air pollutants. A deeper understanding of the effects of various types and characteristics can help guide policymakers in crafting appropriate strategies.

Finally, most studies only focus on the causal relationship between air pollution and mental health, but few attentions have been to explore the level at which AQI and FDI matter. To fill this gap in the literature, we adopt the threshold model. We study that the impact of air pollution on mental health partly depends on the levels of air pollution and the scale of FDI. It is important for local government to set air pollution standards and control the quantity of foreign investment.

This article has been organized in the following ways: Section Literature Review is the literature review. The data and the methodology are introduced in Section Data and Methodology. Section Empirical Results presents the results. Section Discussion discusses the significant findings and the final section concludes.

## Literature Review

The first strand of the literature investigated the relationship between health and air pollution. A large number of studies have examined the effects of air pollution on physical health (7). They found that air pollution could lead to various diseases such as malignant tumor, asthma, lung cancer, and respiratory diseases (21, 22). Among these, the elderly and children are more vulnerable to air pollution (11, 12). In recent years, there has been a surge of interest in the effects of air pollution on mental health (8, 9). Air pollution affects mental health mainly in three ways: First, air pollutants cause depression and neurodegeneration by increasing oxidative stress and cardiac medical conditions in the body (23, 24). Second, air pollution damages physical health and reduces outdoor activities, thus increasing people's loneliness and anxiety. Third, air pollution leads to the loss of human capital, which results in lower income and, ultimately, lower life satisfaction. On empirical side, however, there are some inconsistent findings on the relationship between air pollution and mental health (25). Some found that air pollution has negative effects on mental health through causing depression, restlessness, and stress (21, 26–28). There are possible associations between particulates and suicide, schizophrenia, and psychosis (29–31). The results of Zijlema et al. showed positive relationship between air pollution and mental health (14). The answers to the above questions need to be further studied and judged.

The influence of FDI on the nexus between air pollution and mental health is still uncertain. FDI plays a role mainly through the three channels as follows. (1) Income channel. On one hand, the production process of FDI is increasing returns to scale and FDI will promote economic growth and raise the income level (32). Higher income level leads to higher affordability for health investments, thus improving mental health (33). On the other hand, FDI has a positive effect on wages of skilled workers in the host country, which aggravates the income gap between skilled and unskilled workers (34, 35). This will increase frustration and stress among unskilled workers and make them more easily affected by air pollution; (2) Medical resource channel. FDI can improve the supply of medical services in host countries by directly flowing into the health sector (36). In addition, the increase in FDI helps the local government to increase the revenue intake and invest more medical and health services, which will reduce adverse effects of air pollution on health (37); and (3) Environment channel. Some studies supported the “pollution heaven” theory (38, 39). This implies that low-quality FDI tends to flow to the host country with lower environmental standards, thus leading to deteriorating air quality (40, 41). Others proved the “pollution halo” effects. They argued that FDI could improve air quality through new technologies and green production, which could moderate the negative impact on mental health (42, 43).

## Data and Methodology

### Methodology

#### Main Effects Model

We adopted a panel dataset to consider individual heterogeneity. Therefore, this article uses the fixed effects regression model to study the effects of air pollution on mental health.


(1) $$\begin{array}{r} {mental\;health}_{ijt}=a_{0}+a_{1}{AQI}_{ijt}+a_{2}X_{ijt}+\gamma_{i}+\delta_{t}+\varepsilon_{ijt} \end{array}$$

where *i* represents the individual, *j* is the city, *mental health*_*ijt*_ is the mental health state of *i* who lives in city *j* on year *t*, *AQI*_*ijt*_ is air quality index representing air pollution, *X*_*ijt*_ is control variables influencing mental health at the individual, household, and regional levels, γ_*i*_ is the individual fixed effect, and δ_*t*_ is the time fixed effects.

However, due to the endogeneity caused by reverse causation and measurement error, the effects of air pollution on mental health will be biased. Therefore, we adopt the two-stage least squares (2SLS) for IV estimation. The ventilation coefficient (*VC*) is chosen referring to Broner et al. (18), which is the product of wind speed (*ws*) determining horizontal diffusion rate and atmospheric boundary layer height (*blh*) determining the height which air pollution disperses. On one hand, the *VC* satisfies the correlation requirement. The larger the ventilation coefficient is, the stronger transport and diffusion capacity of air pollutants is, leading to the lower concentration of air pollution. On the other hand, the *VC* is determined by exogenous geographical characteristics. Therefore, the *VC* satisfies requirements of instrumental variables. The IV estimation can be written as follows:


(2) $$\begin{array}{rcl} {AQI}_{ijt} & = & a_{0}+a_{1}{VC}_{ijt}+a_{2}X_{ijt}+\gamma_{i}+\delta_{t}+\varepsilon_{1ijt} \end{array}$$

(3) $$\begin{array}{rcl} {\;mental\;health}_{ijt} & = & a_{0}+a_{1}{\hat{AQI}}_{ijt}+a_{2}X_{ijt}+\gamma_{i}+\delta_{t}+\varepsilon_{2ijt} \end{array}$$

#### Moderation Effect Model

Furthermore, we add the interaction term between FDI and air pollution to study whether FDI has a moderating effect on the nexus between mental health and air pollution as follows:


(4) $$\begin{array}{l} \begin{array}{ccc} mental\;health_{ijt} & = & \beta_{0}+\beta_{1}AQI_{ijt}+\beta_{2}FDI_{ijt}+\beta_{3}AQI_{ijt} \end{array}\\ \;\begin{array}{c} \times \;FDI_{ijt}+\beta_{4}X_{ijt}+\gamma_{i}+\delta_{t}+\varepsilon_{ijt} \end{array} \end{array}$$

where β_3_ represents the extent of moderating effects of *FDI*_*ijt*_.

#### Threshold Effect Model

Some studies have proved the non-linear relationship between FDI and air pollution (44). We further conjecture that the effects of air pollution on mental health could be different when FDI and air pollution are at various levels. We apply the threshold model proposed by Hansen to test the threshold effects of FDI and AQI (45). The threshold estimation model taking FDI as an example is presented as follows:


(5) $$\begin{array}{l} \;mental\;health_{ijt}\\ =\{\frac{\beta_{0}+\beta_{1}AQI_{ijt}+\beta_{2}FDI_{ijt}+\beta_{3}AQI_{ijt}\times \;FDI_{ijt}+\beta_{4}X_{ijt}+\varepsilon_{ijt}\;if\;FDI_{ijt}\leq \tau \;}{\beta_{0}+\gamma_{1}AQI_{ijt}+\beta_{2}FDI_{ijt}+\gamma_{3}AQI_{ijt}\times \;FDI_{ijt}+\beta_{4}X_{ijt}+\varepsilon_{ijt}\;if\;FDI_{ijt}>\tau } \end{array}$$

where *I*(•) is the indicator function.

**Figure 1:**
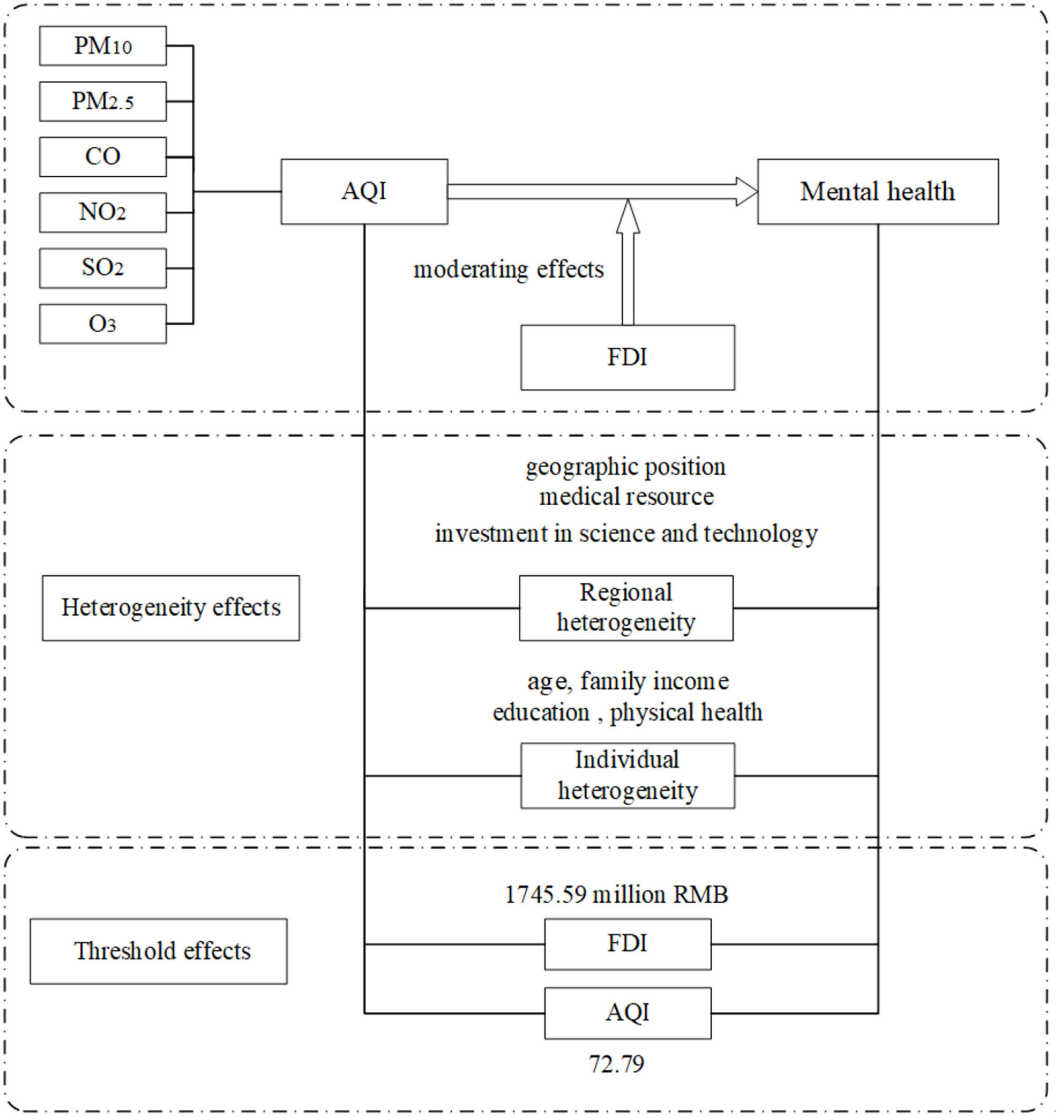
Detailed process of the study.

We determine the number of thresholds based on the minimization of the squared residuals. The details of this article are given in Figure 1.

### Data

The data is from the China Health and Retirement Longitudinal Study (CHARLS) organized by the National School of Development of Peking University. The survey covers ~19,000 residents from 150 county-level units of 28 provinces-level units, reflecting the health, lifestyle, family, and economy of residents. The AQI index is fully available since 2014 and comes from Ministry of Ecology and Environment of the People's Republic of China (https://www.mee.gov.cn/). Therefore, we adopt the 2015 and 2018 CHARLS data. After deleting the missing and abnormal values, 8,992 effective samples aged 45 years or older are finally used. Variables of regional characters are collected from China City Statistical Yearbook.

The explained variable is mental health, including episodic memory, cognitive ability, and depressive symptoms [Center for Epidemiologic Studies-Depression (CES-D)]. We use the entropy weight method to construct the index, which has commonly been used (46). The method could avoid the deviation caused by human factors. We construct the mental health indicator and weights are shown in Table 1. The bigger the variable of mental health, the healthier the people are.

**Table 1:** Construction of mental health index.

**Index**		**Definition**	**Range**	**Weight**
Episodic memory	Short-Term	10 questions: one point for each correct answer	[0, 10]	0.1963
	LONG-TERM	10 questions: one point for each correct answer	[0, 10]	0.2000
Cognitive ability		The number of correct answers questions including calculations, dates, seasons, etc	[0, 10]	0.1986
CES-D	Negative	Questions about respondents' feelings and behavior in the last week	[−32, 0]	0.1976
	Positive		[0, 8]	0.2074

The explanatory variable is air pollution measured by 1-year lagged air quality index (AQI) and six major air pollutants (*PM*_2.5_, *PM*_10_, *CO*, *NO*_2_, *SO*_2_, and *O*_3_). The value of AQI ranges from 0 to 500 and the higher the value, the more serious the air pollution. The moderator is FDI measured by the amount of foreign investment actually used in each city. The value of FDI is converted into renminbi (RMB) using the annual average exchange rate and is adjusted to eliminate the price effect with 2,000 as the base period. The logarithm of FDI, fiscal expenditure, and gross domestic product (GDP) are adopted. GDP and fiscal expenditure are adjusted to eliminate the price effect with 2,000 as the base period. The CHARLS data is matched the air quality and regional variables with the country code. The descriptive statistics and the definition are shown in Table 2. The mean of AQI is 90.78 and the mean FDI is 7,600.06 million RMB. The proportions of males and females are 51 and 49%, respectively. Married people account for mostly 89%. The mean years of education were 8.01. The secondary industry, which is usually energy consuming and pollution intensive, is still the main pillar and accounts for 46.64%.

**Table 2:** Descriptive statistics.

**Variables**	**Definition**	**Mean**	**Std.Dev**	**Min**	**Max**
Mental health	Episodic memory, cognitive ability and depressive symptoms	55.60	15.05	7.81	95.84
AQI	Air quality index	90.78	27.32	46.12	173.11
FDI	Foreign direct investment in a city (million)	7,600.06	13,608.3	30.82	89,354.98
Gender	Male = 1; Female = 0	0.51	0.50	0	1
Age	Age (years)	61.40	8.17	47	84
Rural types	Agriculture hukou = 1; Others = 0	0.62	0.49	0	1
Married	Married = 1; Others = 0	0.89	0.31	0	1
health	Very healthy = 1; health = 2; relatively healthy = 3; general = 4; unhealthy = 5	3.17	0.99	1	5
Education	Years of schooling (years)	8.01	5.22	0	25
Activity	Done voluntary or charity work yes = 1; no = 0	0.02	0.15	0	1
Per income	Personal income (yuan)	11,105.19	20,697.6	0	500,000
All income	Family income (yuan)	26,017.82	88,721.3	−150,000	5,400,000
GDP	GDP of the city (million yuan)	241,000	84,351	2,507.65	661,844
Fiscal exp	Fiscal expenditure (million yuan)	42,589.16	52,359.3	5,586.02	569,785.2
Industry	The proportion of secondary industry (%)	46.64	7.75	19.01	66.26
Medical resource	Doctors per 10,000 people	26.12	10.01	13.11	83.59
Hospital	The number of hospitals	216.68	135.01	31	988

**Figure 2:**
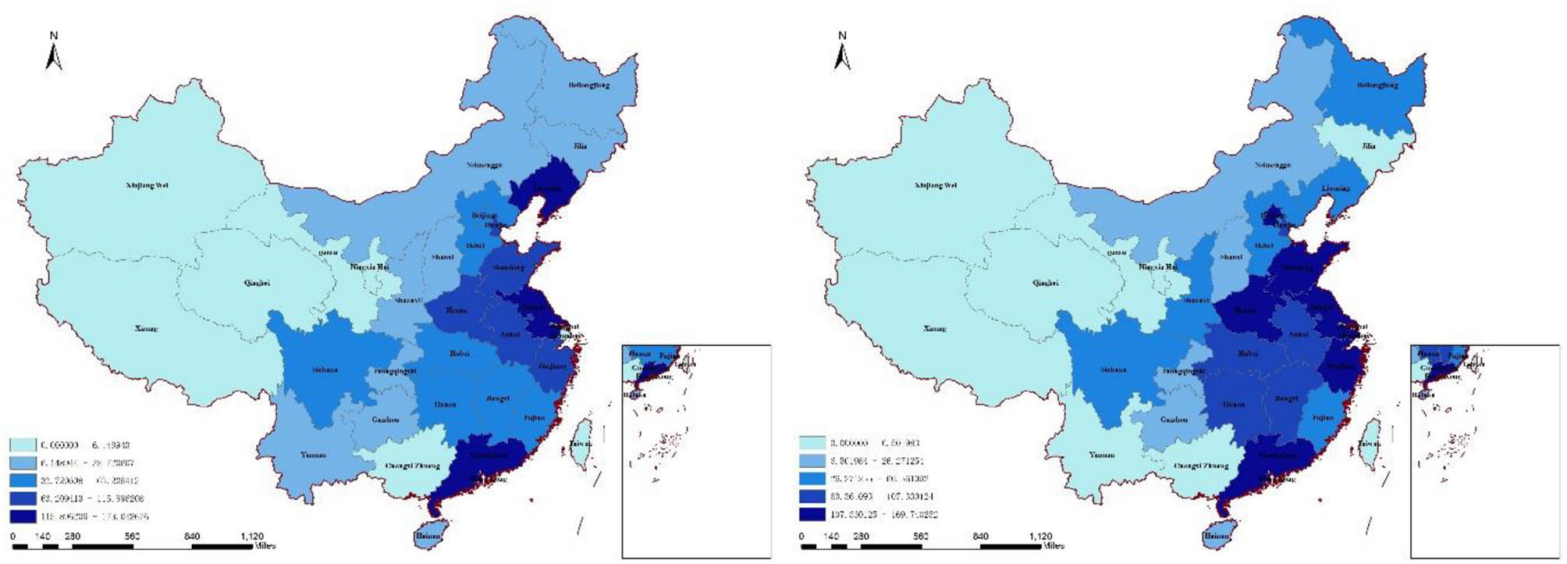
Foreign direct investment (FDI) in 2014 and 2017.

**Figure 3:**
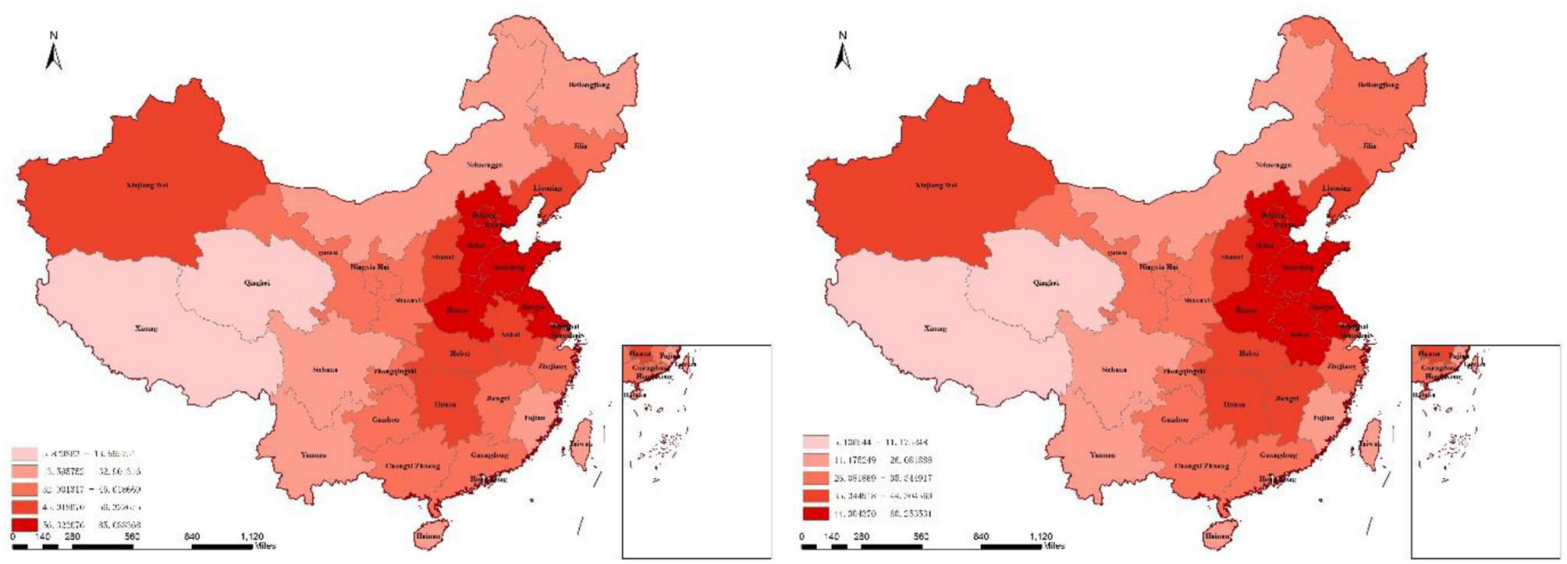
The concentration of *PM*_2.5_ in 2014 and 2017.

Figure 2 presents the distribution of FDI in 2014 and 2017. The FDI is relatively higher in eastern and central regions. It can be seen in Figure 3 that the concentration of *PM*_2.5_ is relatively higher in central regions, while it is relatively lower in southeast coastal regions.

## Empirical Results

### Main Effect and Moderating Effect

The effects of air pollution on mental health and the moderating effects of FDI are shown in Table 3. The results of the fixed effects estimation [models (1) and (3)] represent the negative effect of air pollution on mental health and the moderating effects of FDI. The result of Hausman specification test rejects the null hypothesis that all the variables are exogenous^1^. We further resort to using the ventilation coefficient (*VC*) for an IV estimation, which is supported by the first-stage results. There exists a positive relationship between the *VC* and AQI and the F statistics are considerable. As shown in model (4), when FDI is at the mean level, the impact of air pollution on mental health is 0.371. The coefficient of the interaction term of FDI and air pollution is significantly positive and equal to 5.559. That implies that FDI has an alleviating effect on the negative impacts of air pollution.

**Table 3:** Regression results for the main effect and moderating effect.

**Variable**	**Main effect**			**Moderating effect**		
	**(1) FE**	**(2) IV estimation**		**(3) FE**	**(4) IV estimation**	
		**First stage**	**Second stage**		**First stage**	**Second stage**
AQI	−0.057^***^		−0.684^*^	−0.072^***^		−0.371^**^
	(0.022)		(0.365)	(0.022)		(0.166)
AQI*FDI				1.420^***^		5.559^*^
				(0.495)		(3.274)
VC		16.885^***^			14.012^**^	
		(5.601)			(5.546)	
VC*FDI					4.451^***^	
					(0.497)	
FDI				−0.275	−4.958^**^	−0.818
				(0.424)	(0.4255)	(0.742)
Age	−3.287^***^	−3.501^***^	−5.899^***^	−3.411^***^	−4.974^***^	−4.975^***^
	(0.384)	(0.007)	(1.444)	(0.464)	(0.512)	(0.935)
Education	0.002	−0.317	−0.296	−0.024	−0.227	−0.356
	(0.383)	(0.500)	(0.787)	(0.377)	(0.476)	(0.698)
Married	1.339	−0.550	1.050	1.682	−0.070	2.368
	(2.083)	(1.723)	(2.971)	(2.081)	(1.796)	(2.811)
Per income	0.778^***^	0.262	0.984^***^	0.814^***^	0.307	1.103^***^
	(0.171)	(0.204)	(0.307)	(0.172)	(0.197)	(0.302)
Health	1.071^***^	0.296	1.149^**^	1.071^***^	0.348	1.032^**^
	(0.322)	(0.399)	(0.520)	(0.321)	(0.390)	(0.451)
Activity	2.123	−0.068	1.580	2.009	−0.641	0.991
	(1.407)	(1.696)	(2.310)	(1.391)	(1.567)	(1.983)
Chronic	−0.858^**^	0.840	−0.204	−0.871^**^	0.529	−0.584
	(0.401)	(0.577)	(0.707)	(0.401)	(0.569)	(0.599)
Per GDP	0.191	5.688^***^	4.103^*^	1.398	5.154^***^	6.274^**^
	(0.831)	(0.920)	(2.470)	(0.897)	(0.921)	(3.143)
Industry	−0.183^***^	−0.972^***^	−0.842^**^	−0.265^***^	−1.113^***^	−0.752^***^
	(0.054)	(0.001)	(0.376)	(0.060)	(0.055)	(0.255)
Fiscal exp	4.958	−53.717^***^	−24.069	5.023	−39.260^***^	−4.852
	(3.621)	(4.239)	(19.570)	(3.946)	(4.364)	(9.621)
Medical resource	−0.093	−0.553^***^	−0.600^**^	−0.136	−0.912^***^	−0.533^**^
	(0.094)	(0.135)	(0.279)	(0.096)	(0.155)	(0.213)
Hospital	−0.002	−0.023^***^	−0.023^**^	−0.003	−0.019^***^	−0.015^**^
	(0.003)	(0.003)	(0.010)	(0.003)	(0.003)	(0.006)
Technology	−0.874	7.495^***^	3.695	0.116	8.969^***^	4.913^*^
	(0.883)	(0.773)	(2.805)	(0.944)	(0.784)	(2.856)
**Fixed effects**
Individual	Yes	Yes	Yes	Yes	Yes	Yes
Year	Yes	Yes	Yes	Yes	Yes	Yes
F statistics	57.19	374.92		65.59	314.45	
	0.1439	0.7501		0.1478	0.7607	
*N*	6,729	4,836	4,836	6,729	4,836	4,836

^***^, ^**^, and ^*^, respectively, indicate significance at the 1, 5, and 10 levels.

Table 3 also reports the impacts of control variables on mental health. It can be observed that people with higher personal income have better mental health. Older people are associated with lower mental health index (column 2 in model 4). People with better health are associated with a decrease of 1.032 in mental health. Moreover, cities with higher GDP and investment in science and technology will be beneficial for people's mental health.

Furthermore, we replace AQI with the concentration of *PM*_2.5_, *PM*_10_, *CO*, *NO*_2_, *SO*_2_, and *O*_3_ to study the different impacts of the six air pollutants on mental health. It can be observed in Table 4 that the effects vary by the type of air pollution. *PM*_2.5_, *PM*_10_, *SO*_2_, *CO*, and *NO*_2_ have negative impacts on mental health at the 5% level of significance, while the effects of *O*_3_ are not significant. In addition, the estimated coefficients of the interaction term of FDI and *PM*_2.5_ and *PM*_10_ are 1.789 and 2.224, all of which are significant. The moderating effects of FDI are more obvious on particulate matter.

**Table 4:** Regression results of six major air pollutants as the independent variables.

	**Main effect**	**Moderating effect**		**Main effect**	**Moderating effect**
**PM**_**2.5**_			**NO**_**2**_		
PM_2.5_	−0.546^**^	−0.385^*^	NO_2_	−2.296^*^	−2.353
	(0.002)	(0.197)		(1.299)	(1.518)
FDI		−1.237^*^	FDI		−1.263
		(0.656)			(1.004)
${\text{PM}}_{2.5}^{*}$FDI		1.789^**^	${\text{NO}}_{2}^{*}$FDI		−0.670
		(0.779)			(1.783)
Control variable	Yes	Yes	Yes	Yes	Yes
Fixed effects	Yes	Yes	Yes	Yes	Yes
*N*	4,836	4,836		4,836	4,836
**PM**_**10**_			**CO**		
PM_10_	−0.376^*^	−0.384^**^	**CO**	−23.305^**^	−25.106^**^
	(0.192)	(0.189)		(10.854)	(11.337)
FDI		−0.0004	FDI		1.171
		(0.0004)			(1.170)
${\text{PM}}_{10}^{*}$FDI		2.224^*^	${\text{NO}}_{2}^{*}$FDI		−0.440
		(1.148)			(0.580)
Control variable	Yes	Yes	Yes	Yes	Yes
Fixed effects	Yes	Yes	Yes	Yes	Yes
*N*	4,836	4,836		4,836	4,836
SO_2_			**O**_**3**_		
SO_2_	−0.307^**^	−0.313^**^	O_3_	1.147	1.254
	(0.142)	(0.144)		(1.024)	(1.136)
FDI		−0.0003	FDI		−4.504
		(0.0004)			(3.897)
${\text{SO}}_{2}^{*}$FDI		0.547	${\text{O}}_{3}^{*}$FDI		2.115
		(0.565)			(1.765)
Control variable	Yes	Yes	Yes	Yes	Yes
Fixed effects	Yes	Yes	Yes	Yes	Yes
*N*	4,836	4,836		4,836	4,836

^**^ and ^*^, respectively, indicate significance at the 1, 5, and 10 levels.

### Robustness Test

In this section, we test the robustness of our main results by three methods. First, we use the variable whether participating in social activities such as dancing and sports to represent mental health. The reason is that people with better mental health are likely to go to sport, social, or other clubs. Air pollution is harmful to physical health and reduces outdoor activities, which could damage mental health in the long term. Second, we replace the mental health index with negative emotions^2^. Third, we delete a part of independent variables. Local governments may falsify the AQI index because environmental indicators such as AQI are included in performance assessment. The qualified AQI indicates its value <100 and AQI forgery usually does not deviate too far from the truth. Therefore, we delete the values of AQI index between 99 and 100, which are likely to be tampered. It can be seen in Table 5 that air pollution negatively affects mental health and FDI has an alleviating influence on the relationship between air pollution and mental health. Our results are robust.

**Table 5:** Robustness test.

	**Robustness test 1 entertainment**		**Robustness test 2 Negative emotions**		**Robustness test 3 deleting AQI 99–101**	
AQI	−0.022^*^	−0.014^**^	0.224^*^	0.097	−0.523^*^	−0.494^*^
	(0.013)	(0.006)	(0.135)	(0.062)	(0.309)	(0.277)
FDI		−0.002		−0.137		−2.485^**^
		(0.019)		(0.308)		(0.980)
AQI*FDI		0.151^*^		−2.349^*^		2.140^*^
		(0.088)		(1.265)		(1.202)
Control variable	Yes	Yes	Yes	Yes	Yes	Yes
Fixed effects	Yes	Yes	Yes	Yes	Yes	Yes
*N*	4,836	4,836	4,836	4,836	4,523	4,523

^**^ and ^*^, respectively, indicate significance at the 1, 5, and 10 levels.

### Heterogeneity Test

This section discerns the effects of air pollution and FDI among the groups with different individual and regional characteristics. Tables 6, 7 show regional heterogeneity based on the difference of location, medical resource, and investment in science and technology. Table 8 reports individual heterogeneity based on education, age, family income, and physical health difference.

**Table 6:** Heterogeneity in the eastern, western, and central regions.

	**Region**					
	**Eastern**		**Central**		**Western**	
AQI	−0.086^***^	−0.098^***^	0.033	−0.023	−0.015	0.055
	(0.019)	(0.019)	(0.161)	(0.170)	(0.080)	(0.143)
FDI		−0.494		−2.765		−0.630
		(0.421)		(3.185)		(1.178)
AQI*FDI		1.605^***^		−0.760		2.264
		(0.434)		(1.933)		(3.596)
Control variable	Yes	Yes	Yes	Yes	Yes	Yes
Fixed effects	Yes	Yes	Yes	Yes	Yes	Yes
*N*	5,495	5,495	1,914	1,914	1,546	1,546

^***^, respectively, indicate significance at the 1, 5, and 10 levels.

**Table 7:** Heterogeneity based on medical resource and investment in science and technology.

**Medical resources**				
**Variables**	**Relative abundance**		**Relative lack**	
AQI	−6.552	−0.372	−0.269^*^	−0.268^*^
	(9.927)	(0.230)	(0.163)	(0.160)
FDI		−1.696		−0.589
		(1.543)		(0.874)
AQI*FDI		4.640^**^		1.320
		(2.363)		(1.123)
*N*	1,763	1,763	3,073	3,073
**Government investment in science and technology**				
**Variables**	**More investment**		**Less investment**	
AQI	2.543^**^	−0.600	−0.761^*^	0.035
	(0.754)	(0.778)	(0.394)	(0.250)
FDI		−19.863^***^		5.761
		(7.227)		(4.868)
AQI*FDI		27.450^***^		19.985
		(8.602)		(12.997)
*N*	758	758	4,078	4,078
Fixed effects	Yes	Yes	Yes	Yes
Control variable	Yes	Yes	Yes	Yes

^***^, ^**^, and ^*^, respectively, indicate significance at the 1, 5, and 10 levels.

**Table 8:** Heterogeneity based on education, age, income, and changes of physical health.

**Education level**				
**Variables**	**High-Educated**		**Low-Educated**	
AQI	0.047	0.061	−0.824^*^	−0.422^**^
	(0.329)	(0.384)	(0.003)	(0.182)
FDI		2.215		−0.929
		(5.160)		(0.752)
AQI*FDI		7.473		5.851^*^
		(15.966)		(3.315)
*N*	239	239	4,597	4,597
**Age**				
**Variables**	**<60 years**		**>** **or** **=60 years**	
AQI	−1.044	−0.303	−1.074^*^	−0.654^**^
	(1.001)	(0.254)	(0.637)	(0.319)
FDI		5.582		−0.475
		(4.614)		(1.605)
AQI*FDI		−0.662		15.567^**^
		(1.106)		(7.290)
*N*	2,393	2,393	2,220	2,220
**Family income**				
**Variables**	**High-Income**		**Low-Income**	
AQI	−0.037^*^	−0.047^**^	0.004	−0.040
	(0.021)	(0.021)	(0.068)	(0.067)
FDI		−0.151		−0.525
		(0.362)		(1.227)
AQI*FDI		0.864^**^		3.009^**^
		(0.432)		(1.400)
*N*	6,871	6,871	2,084	2,084
**Physical health**				
**Variables**	**Better**		**Worse**	
AQI	−0.039^**^	−0.053^***^	−0.262^***^	−0.300^***^
	(0.019)	(0.019)	(0.059)	(0.061)
FDI		−0.589^*^		−3.331^**^
		(0.327)		(1.444)
AQI*FDI		0.988^***^		1.665
		(0.360)		(1.609)
*N*	7,062	7,062	1,353	1,353
Fixed effects	Yes	Yes	Yes	Yes
Control variable	Yes	Yes	Yes	Yes

^***^, ^**^, and ^*^, respectively, indicate significance at the 1, 5, and 10 levels.

#### Regional Heterogeneity

We first examine the influence across three regions: eastern, western, and central regions. It can be observed in Table 6 that the negative effect of air pollution is significant for people in eastern region, as one rise in AQI index causes an 0.086 (column 1 in Table 6) decrease in mental health. Besides, FDI reduces the harmful impact of air pollution and the coefficient of the interaction term is equal to 1.605. In contrast, air pollution and FDI have no significant effects on mental health in central and western regions.

We divide the sample into the two groups according to medical resources: “relative abundance” and “relative lack.” “Relative abundance” is defined as the cities' hospitals per 10,000 population that are higher than the average for all the cities. Table 7 shows that the effect of air pollution on people living in cities with less medical resources is significantly negative and equal to −0.269. FDI plays a moderating role in cities with abundant medical resources. We further define “more investment” as the region's investment in science and technology above the average, while the cities below the average are “less investment.” It can be observed that people in the “less investment” group suffer from air pollution. FDI moderates the adverse impact of air pollution on people living in cities with more investment in science and technology.

#### Individual Heterogeneity

In this section, we divide the samples according to the education level, age, income, and changes of physical health. Table 8 summarizes the estimated results. First, low-educated people are defined who did not enter university (<16 years); otherwise, people are categorized as the high educated. It can be observed that air pollution has a negative impact on mental health of low-educated people and high-educated people are less affected by air pollution. Moreover, the moderating effect of FDI is more related to low-educated people. Second, we further study the age difference in response to air pollution and set 60 years old as the classification standard. Older people are more vulnerable to air pollution and FDI only moderates the adverse impact of air pollution on older people's mental health. Third, the samples' family income level below the average is defined as “low income,” while higher than the average income level is defined as “high income.” Compared with the low-income groups, air pollution has a significant impact on mental health of high-income people. The coefficient of the interaction term of FDI and air pollution is significant and equal to 3.009, implying that FDI could alleviate harmful effects of air pollution for low-income people. Finally, we divide samples into the two groups according to the changes of self-rated health level. Mental health of people whose physical health gets worse is more susceptible to air pollution. FDI could alleviate the adverse impact of air pollution on people whose physical health becomes better.

### Threshold Effects

Table 9 summarizes the threshold effects of FDI and AQI, showing the effects of different levels of AQI and FDI. The F statistics of threshold effect test on AQI and FDI is 22.11 and 21.41, which verify the threshold effects. The threshold value of FDI is 1,745.591 million RMB. This result shows that FDI more than the threshold value will insignificantly affect the negative effect of air pollution on mental health. FDI has an alleviating effect on the harmful effects of air pollution when the FDI is >1,745.591 million RMB. There are 11.19% of cities in our sample exceed the threshold value and mainly belong to the eastern region. Second, the threshold value of AQI is 92.79, which is lower than the qualified AQI (100) in China. When the value of AQI exceeds this threshold, the coefficient of the effects of air pollution decreases from −0.045 to −0.06. Only if AQI is <92.79, the moderating effect of FDI is significantly positive and equal to 2.172.

**Table 9:** Regression results of the threshold model.

		**AQI**	AQI*FDI
	**Percentage**	**The threshold effect of FDI**	
FDI **≤** 1,745.59	88.81%	−0.076^***^ (0.016)	0.449 (0.447)
FDI **>** 1,745.59	11.19%	−0.056^***^ (0.018)	1.110^**^ (0.497)
Control variables		Yes	Yes
Fixed effects		Yes	Yes
	**Percentage**	**The threshold effect of AQI**	
AQI **≤** 92.79	54.41%	−0.045^**^ (0.019)	2.172^***^ (0.469)
AQI **>** 92.79	45.59%	−0.060^***^ (0.016)	0.461 (0.522)
Control variables		Yes	Yes
Fixed effects		Yes	Yes

^***^ and ^**^ respectively, indicate significance at the 1, 5, and 10 levels.

## Discussion

Based on the above results, air pollution has a negative effect on mental health, which confirms the finding of Shen et al. (7). Air pollutants cause anxiety and depression symptoms (47, 48) by increasing oxidative stress and cardiac medical conditions in the body (23, 24, 49). Air pollution also reduces outdoor activities, therefore increasing people's loneliness (50). In addition, air pollution causes the loss of human capital, which leads to lower income and ultimately reduces our happiness. The results also support that FDI dilutes the adverse impacts of air pollution on mental health (51). On one hand, FDI may increase people and government's investment in mental health through promoting economic growth and arousing the awareness against pollution (52). Higher income level leads to higher affordability for health investments for residents and government, thus improving mental health. On the other hand, China's government has given priority to protect environment and encouraged more FDI enterprises to the high-tech industry in recent years. The role of FDI in China supports the “pollution halo” hypothesis, which will also help to alleviate the effect of air pollution.

There are differences in the influence of different types of pollutants on mental health. Among the six kinds of air pollutants, *PM*_2.5_, *PM*_10_, *CO*, *NO*_2_, and *SO*_2_ have a significantly negative impact on the mental health. This result may be due to the different nature and concentration of these pollutants in China. Particulate matter is the one among the most ubiquitous pollutant deteriorating the air quality (29, 53, 54). *PM*_2.5_ and *PM*_10_ are not only harmful to human health such as chronic problems, but also play a vital role in haze and climate change. *CO* and *NO*_2_ could damage physical health, in particular, respiratory diseases (55) and cardiovascular diseases (56, 57). High concentrations of *CO* and *NO*_2_ are linked to cancer and death (58). Long-term exposure to *CO* and *NO*_2_ will reduce ability to work and decrease human capital. Lower income and depression are caused by air pollution that could harm to mental health. Results reflect those of Chen et al. (59) and O'Neill et al. (60) who also found that higher concentration of *PM*_2.5_ lead to mental illnesses. *O*_3_ has no significant effect on mental health, which is contrary to some previous studies (61). This inconsistency may be due to the difference of the amount and time of exposure, the accumulation of *O*_3_, study designs, and definition of mental health (25). In addition, FDI has moderating effects on the relationship between mental health and *PM*_2.5_ and *PM*_10_. Anthropogenic activities, which are regarded as the main source of particulate matter, include combustion of fossil fuels and industrial emissions. FDI with the advanced antipollution technology can benefit the environment and reduce the harmful impact of mental health.

Regarding region differences, it is found that only in eastern region, air pollution has a negative effect on mental health, which is also confirmed in Figure 2. People in eastern region are exposed to higher levels of particulate matter. The moderating effect of FDI is also obvious. A possible explanation may be that cities in eastern region have higher levels of economic development and better infrastructure. Therefore, the government is more likely to invest in healthcare (52). Opening health sector to international participation would also enable to reduce adverse effects of air pollution (36).

The moderating effects of FDI are significant in cities with abundant medical resource. The result validates the way that FDI plays a moderating role, i.e., FDI can improve the supply of medical services and help the local government to invest more medical resources (36). Regarding the difference of the investment in science and technology, the above results imply that cities with more attention to science and technology tend to attract FDI with more advanced technology and lower pollutant emissions. The green investment and production will improve air quality and mental health. However, cities with less investment in technology still rely on heavy industry. Foreign-funded projects in these cities are mainly pollution intensive and less involved in health sectors.

The mental health of people who are elder, low educated, high income, or getting worse physical health are more susceptible to air pollution. FDI also has a moderating effect on mental health of older, low-educated, or more unhealthy people. There are several possible explanations. Compared with low-educated people, high-educated people learn more environmental knowledge and air pollution and they have more strategies to self-protect against pollution. Second, the effects of air pollution and FDI are more significant to the low-income group (4). The high-income people generally have a better understanding of air pollutants and take some preventive actions for self-preservation. Therefore, FDI has higher marginal benefits to low-income people. Finally, people whose health gets worse are more vulnerable to air pollution. They have stronger concern about the harm for their health when facing up to pollution, thus may lead to depression, helplessness, and stress. FDI can dilute the adverse effects of air pollution on people with better physical fitness because they are more energetic to make use of resources from FDI.

The threshold effect adds further insight of the effects of different levels of FDI and AQI. FDI causes a reduction in the negative relationship between air pollution and mental health, when the FDI is >1,745.591 million RMB. When FDI scale is low, local governments have a strong incentive to introduce foreign investment to promote economic development with lower environmental standards. These will ignore health guidance and investment and lead to the increase of air pollutants (62, 63). As increase in FDI and income level, the attention shifts to protect environment, take actions to reduce harmful effects of air pollution, and improve mental health. There are still 88.81% of cities' FDI lower than the threshold. It is worth noting that the air pollution has exceed the threshold value in about half of the cities where the role of FDI is not significant.

Our conclusions have some limitations. First, although AQI includes six major air pollutants, it cannot sufficiently study the impact of air pollution, especially the effects of total number of pollutants. Second, due to the limitation of data, this study cannot cover the impact of COVID-19 outbreak. COVID-19 outbreak may alter the supply and demand of FDI and directly affect mental health and the attitude to air pollution. Third, the CHARLS opens its data on the location only in the city level. Therefore, we use city-wide average air pollution concentrations rather than personal exposure measures, which may not precisely reflect the effects of air pollution on mental health.

## Conclusion and Policy Implications

This article examines the relationship between air pollution and mental health and the moderating role of FDI based on the CHARLS data in 2015 and 2018 using the IV fixed effects model and threshold model. First, air pollution has negative effects on mental health. Among the six types of air pollution, *PM*_2.5_, *PM*_10_, *SO*_2_, *CO*, and *NO*_2_ have significantly negative impacts on mental health. Second, FDI has an alleviating influence on the negative relationship between air pollution and mental health. Third, the regional heterogeneity shows that the negative effect of air pollution and the moderating effect of FDI are significant for people in eastern region. FDI positively affects people living in cities with more investment in science and technology and abundant medical resources. In addition, the individual heterogeneity results show that air pollution has more heavily negative impacts on people who are elder, low educated, high income, or unhealthy. Finally, FDI moderates the adverse impact between air pollution and mental health when it is >1,745.591 million RMB. Air pollution has larger negative effects on mental health when the value of AQI is >92.79. There are only 11.19% of cities in our sample that exceed the threshold value in China. Therefore, how to attract and use FDI effectively to moderate the relationship between air pollution and mental health for governments and enterprises could also be a far-reaching direction for future studies.

The above findings have a number of practical implications for policymakers. First, it is important to pay particular attention to the effects of air pollution not only on the physical health, but also on mental health. Policymakers need to explicitly consider the effects on mental health when formulating environmental protection policy, as well as policy evaluation. Second, the “polluted heaven” hypothesis for air pollution is not valid in China. An important policy priority should, therefore, be to provide subsidies or finance support to encourage businesses to receive high-quality foreign investment. In addition, the government should control and guide the scale of FDI and the level of air pollution according to the threshold value. Fourth, the effects of different types of air pollutants should be fully recognized. There should be a greater focus placed on *PM*_2.5_, *PM*_10_, *SO*_2_, *CO*, and *NO*_2_ when government crafts appropriate strategies to reduce air pollution. Finally, the findings of regional heterogeneity suggest that government should actively guide FDI to the central and western region. Cities with less investment in technology should focus on improving their own absorption capacity of FDI and make full use of new technology and experience brought by FDI. Moreover, air pollution and FDI disproportionally affect those who are low education, low income, or older stresses. Therefore, related policies cannot only bring mental health benefits as a whole, but also result in an equitable distribution. There should be improving education level and providing targeted protection and advice to people who are susceptible to air pollution.

## Data Availability Statement

The original contributions presented in the study are included in the article/Supplementary Material, further inquiries can be directed to the corresponding author/s.

## Author Contributions

WJ: conceptualization, methodology, writing—reviewing and editing, and supervision. YC: software, data curation, writing—original draft, and visualization. Both authors contributed to the article and approved the submitted version.

## Funding

This study was supported by the National Social Science Fund of China (20BJL020).

## Conflict of Interest

The authors declare that the research was conducted in the absence of any commercial or financial relationships that could be construed as a potential conflict of interest.

## Publisher's Note

All claims expressed in this article are solely those of the authors and do not necessarily represent those of their affiliated organizations, or those of the publisher, the editors and the reviewers. Any product that may be evaluated in this article, or claim that may be made by its manufacturer, is not guaranteed or endorsed by the publisher.

## Footnotes

^1^The result of Hausman specification test is 92.71 and rejects the null hypothesis that all the variables are exogenous at the 1% level of significance.

^2^In the CHARLS questionnaire, there are eight questions in CES-D referring to how people have felt and behaved negatively during the last week.
